# CENCAT enables immunometabolic profiling by measuring protein synthesis via bioorthogonal noncanonical amino acid tagging

**DOI:** 10.1016/j.crmeth.2024.100883

**Published:** 2024-10-21

**Authors:** Frank Vrieling, Hendrik J.P. van der Zande, Britta Naus, Lisa Smeehuijzen, Julia I.P. van Heck, Bob J. Ignacio, Kimberly M. Bonger, Jan Van den Bossche, Sander Kersten, Rinke Stienstra

**Affiliations:** 1 Division of Human Nutrition and Health, Wageningen University, Wageningen, the Netherlands; 2 Department of Synthetic Organic Chemistry, Chemical Biology Lab, Radboud University, Heyendaalseweg 135, 6525 AJ Nijmegen, the Netherlands; 3 Department of Molecular Cell Biology and Immunology, Amsterdam Cardiovascular Sciences, Amsterdam Gastroenterology Endocrinology Metabolism, Amsterdam Institute for Infection and Immunity, Amsterdam UMC, Vrije Universiteit Amsterdam, Amsterdam, the Netherlands; 4 Department of Internal Medicine, Radboud University Medical Center, Nijmegen, the Netherlands

**Keywords:** immunometabolism, SCENITH, energy metabolism, glycolysis, OXPHOS

## Abstract

Cellular energy metabolism significantly contributes to immune cell function. To further advance immunometabolic research, novel methods to study the metabolism of immune cells in complex samples are required. Here, we introduce CENCAT (cellular energetics through noncanonical amino acid tagging). This technique utilizes click labeling of alkyne-bearing noncanonical amino acids to measure protein synthesis inhibition as a proxy for metabolic activity. CENCAT successfully reproduced known metabolic signatures of lipopolysaccharide (LPS)/interferon (IFN)γ and interleukin (IL)-4 activation in human primary macrophages. Application of CENCAT in peripheral blood mononuclear cells revealed diverse metabolic rewiring upon stimulation with different activators. Finally, CENCAT was used to analyze the cellular metabolism of murine tissue-resident immune cells from various organs. Tissue-specific clustering was observed based on metabolic profiles, likely driven by microenvironmental priming. In conclusion, CENCAT offers valuable insights into immune cell metabolic responses, presenting a powerful platform for studying cellular metabolism in complex samples and tissues in both humans and mice.

## Highlights

• Immunometabolic profiling of complex samples and tissue-resident immune cells
• Utilizes ncAAs to measure protein translation inhibition without toxic side effects
• CENCAT reproduces known metabolic signatures of macrophage activation
• βES is the preferred ncAA for CENCAT due to its superior incorporation rate

## Motivation

Extracellular flux (XF) analysis has been a key technique in immunometabolism research, measuring the cellular oxygen consumption rate (OCR) and extracellular acidification rate (ECAR) to determine immune cell metabolic profiles. However, XF analysis has several limitations, including the need for purified cells, relatively high cell numbers, and specialized equipment. Recently, a novel flow-cytometry-based technique called SCENITH (single-cell energetic metabolism by profiling translation inhibition) was introduced, which measures the inhibition of cellular protein synthesis as a proxy for metabolic activity in single cells. A limitation of this technique is its reliance on fluorescent staining of intracellular puromycin, a toxic antibiotic that could cause unwanted effects during the assay. To address this, we propose an alternative approach using biorthogonal noncanonical amino acid tagging (BONCAT) to measure protein synthesis.

## Graphical abstract

**Figure:**
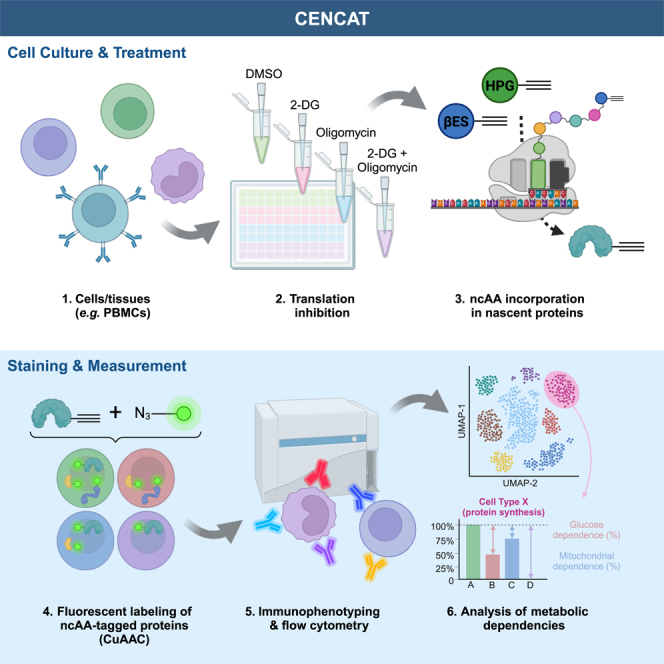

## Teaser

Vrieling et al. present CENCAT, a technique to perform in-depth immunometabolic phenotyping in complex samples. They utilize fluorescent labeling of non-toxic unnatural amino acids to determine cellular dependence on glucose and mitochondrial metabolism for protein synthesis. CENCAT provides a powerful platform for studying cellular metabolism in both humans and mice.

## Introduction

The expanding field of immunometabolism has demonstrated that the functional properties of immune cells are dependent on their metabolic profile. When immune cells are activated, their energy demand increases to support proliferation and effector functions such as the production of cytokines and antibodies. This process involves a significant reprogramming of cellular metabolic pathways. Upon activation, many immune cell types switch from oxidative mitochondrial metabolism to glycolysis, a phenomenon known as the “Warburg effect,” as it was first described in tumor cells by Otto Warburg.^1^ Although essential for immune function, alterations in immune cell metabolism have been associated with various pathologies. For example, chronic low-grade inflammation in adipose tissue is linked to metabolic rewiring of adipose tissue macrophages in obesity,^2^ while “primed” glycolytic inflammatory monocytes are associated with atherosclerosis.^3^ In tumors, immune cell metabolism is reprogrammed by limitations in nutrient availability and the accumulation of metabolic byproducts such as lactate, hindering their effector functions.^4^ Hence, technologies that enable detailed analysis of cellular metabolism are essential for studying the underlying mechanisms of inflammatory diseases.

Seahorse extracellular flux (XF) analysis has been instrumental in making many discoveries in the immunometabolism field. This technique measures the cellular oxygen consumption rate (OCR) and extracellular acidification rate (ECAR) to determine functional metabolic profiles. However, this method has limitations, including its reliance on purified cells, relatively high cell numbers, and specialized equipment and reagents. With the advent of single-cell technologies, developing novel methods to determine functional metabolic profiles of immune cells in complex samples is of great importance. A paper by Argüello et al. recently introduced a technique called SCENITH (single-cell energetic metabolism by profiling translation inhibition). The core principle of SCENITH is that the rate of cellular protein synthesis is a surrogate for metabolic activity and can be used to determine cellular metabolic dependencies. In SCENITH, the incorporation of the antibiotic puromycin into nascent proteins is measured through immunostaining as a proxy for protein synthesis under conditions of metabolic inhibition (2-deoxy-D-glucose [2-DG] to inhibit glucose metabolism, oligomycin to inhibit mitochondrial ATP synthesis), allowing for the determination of cellular dependence on glucose and mitochondrial metabolism. Since SCENITH is a flow-cytometry-based assay, it offers single-cell resolution and requires significantly fewer cells than Seahorse XF analysis. This method has been compared and validated against Seahorse XF analysis^5^^,^^6^ and is thus a promising tool for determining functional metabolic profiles of immune cells in complex samples.

A significant limitation of using puromycin as a proxy for protein synthesis is its inherent toxicity, as it can induce endoplasmic reticulum (ER) stress^7^ and other toxic effects.^8^ Additionally, puromycin was found to not accurately measure protein translation under energy-starved conditions such as 2-DG treatment in some cell types, which could be problematic for its application in SCENITH.^9^ Therefore, we propose an alternative way of measuring protein synthesis in SCENITH through biorthogonal noncanonical amino acid tagging (BONCAT).^10^ In BONCAT, cells are incubated with a noncanonical amino acid (ncAA) analog containing a minimal chemical modification, which can subsequently be targeted to fluorescently tag nascent proteins through copper(I)-catalyzed azide-alkyne cycloaddition (CuAAC),^11^ more commonly known as the “click” reaction. A significant advantage of employing BONCAT for measuring protein synthesis is that ncAAs are non-toxic.^12^ The most widely used ncAAs are two methionine analogs: the azide-bearing azidohomoalanine (AHA) and the alkyne-bearing homopropargylglycine (HPG). As these require depletion of endogenous methionine for adequate protein labeling, a threonine ncAA analog, β-ethynylserine (βES), was recently developed to be able to tag proteins in a complete growth medium.^13^ Among other uses, BONCAT has been used for affinity purification of nascent proteins,^14^ to track and localize the newly synthesized proteins in cells,^15^ and to measure SLC1A5-mediated amino acid uptake.^16^

**Figure 1:**
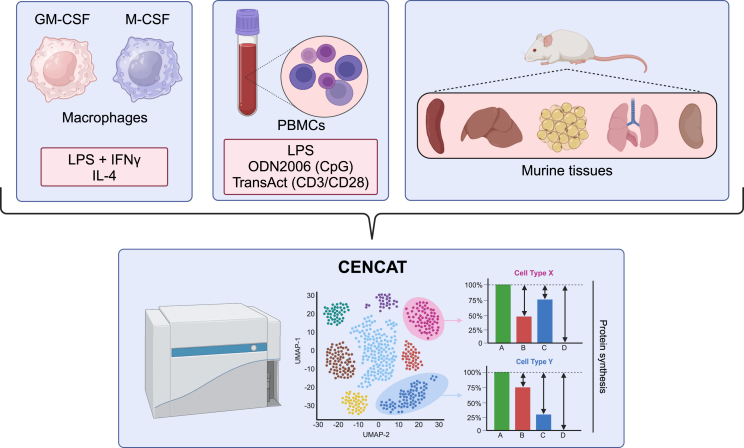
Overview of performed experiments

Here, we investigated the potential of BONCAT using HPG and βES for measuring metabolic dependencies in multiple sample types in a “one-size-fits-all” format, an approach we have termed CENCAT (cellular energetics through noncanonical amino acid tagging). We first show that cellular protein synthesis results in HPG accumulation over time. We subsequently validate our method compared to the original SCENITH protocol in a primary macrophage model of classical vs. alternative activation. Next, we tested CENCAT using HPG or βES for metabolic profiling of peripheral blood mononuclear cells (PBMCs) under different stimulatory conditions. Finally, we demonstrate the technique’s applicability in analyzing tissue-resident immune cell metabolism in mice (Figure 1).

## Results

### CENCAT: Assay concept

After sample workup and plating (1), cells are pre-incubated with DMSO as control, 2-DG to inhibit glucose metabolism, oligomycin to inhibit mitochondrial respiration, or the compounds in combination to block ATP production completely (2). An alkyne-bearing ncAA is subsequently added as a substrate for protein synthesis (3). Cells are then fixed and permeabilized, after which intracellular ncAA-tagged proteins can be labeled through a click reaction with a fluorescent azide probe (4) to be measured by flow cytometry (5). The relative cellular metabolic dependence on glucose and mitochondrial respiration is calculated by comparing ncAA mean fluorescence intensities (MFIs) between the different inhibitor conditions (6). Glucose dependence (%) is calculated by dividing the MFI difference (ΔMFI) between DMSO and 2-DG by the ΔMFI between DMSO and 2-DG + oligomycin. After subtracting the glucose dependence from 100%, the remainder gives you the fatty acid/amino acid oxidation (FAO/AAO) capacity (%). Mitochondrial dependence (%) is the ΔMFI between DMSO and oligomycin divided by the ΔMFI between DMSO and 2-DG + oligomycin. After subtracting the mitochondrial dependence from 100%, the remainder is the glycolytic capacity (%).

### HPG incorporation is a proxy of protein synthesis and can be used as an alternative to puromycin

**Figure 2:**
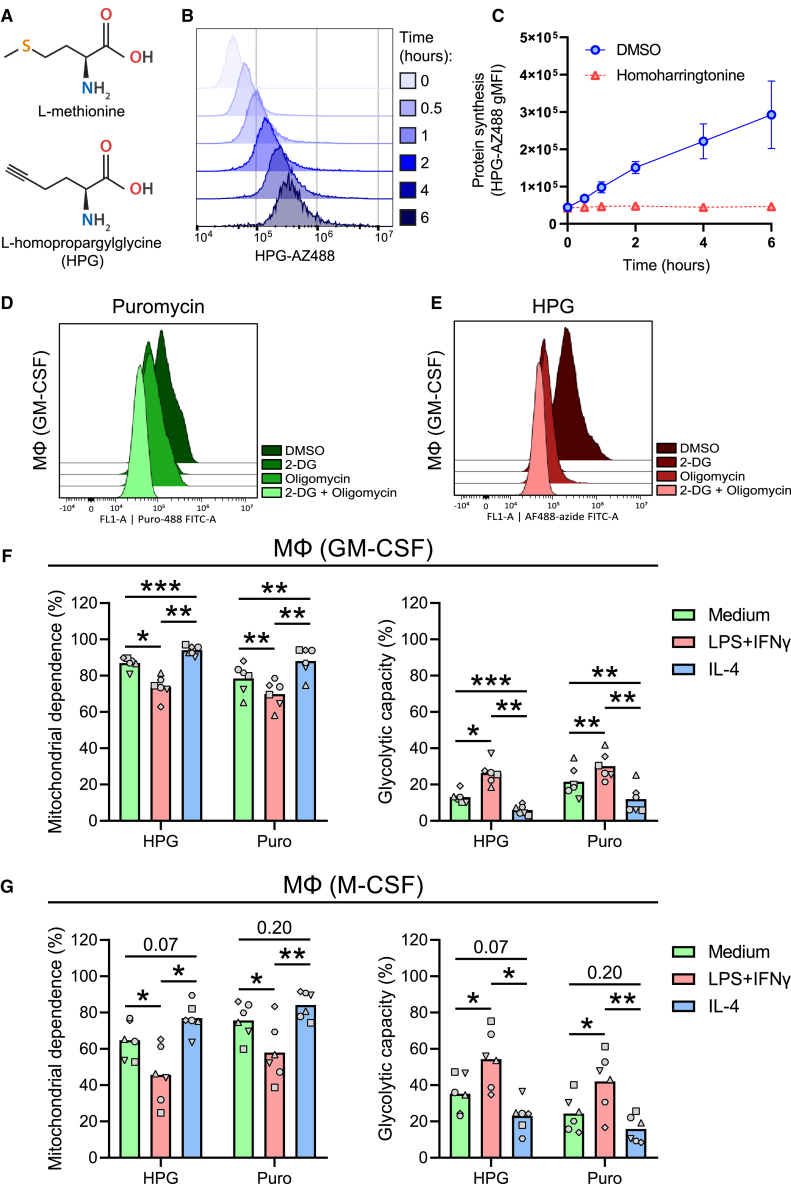
Comparison of CENCAT with the original SCENITH protocol (A) Structures of L-methionine and HPG. (B and C) Primary human macrophages (M-CSF) were treated with HPG and harvested at different time points for click labeling. (B) Representative flow cytometry histograms of HPG-AZ488 staining at 0, 0.5, 1, 2, 4, and 6 h post-treatment. (C) HPG incorporation kinetics of macrophages treated with homoharringtonine (red) or DMSO control (blue). Data are displayed as means ± SD of the geometric MFI (gMFI; *n* = 3). (D and E) Representative histograms of protein synthesis for DMSO, 2-DG, oligomycin, and 2-DG + oligomycin conditions as measured using puromycin (Puro) (D) or HPG (E) in GM-CSF macrophages. (F and G) Primary human macrophages (GM-CSF and M-CSF) were stimulated for 24 h with culture medium (green), LPS + IFNγ (red), or IL-4 (blue) before SCENITH analysis using either HPG or Puro as substrates. (F) Mitochondrial dependence (%) and glycolytic capacity (%) of GM-CSF macrophages. (G) Mitochondrial dependence (%) and glycolytic capacity (%) of M-CSF macrophages. Data are displayed as mean percentages ±SD (*n* = 6). Significance was tested by two-way ANOVA with Sidak correction for multiple testing. Individual donors are displayed by different symbols. ∗*p* < 0.05, ∗∗*p* < 0.01, and ∗∗∗*p* < 0.001.

HPG is an alkyne-bearing methionine analog (Figure 2A) previously used to tag nascent proteins.^14^ To validate whether HPG incorporation in immune cells reflects protein synthesis, macrophage colony-stimulating factor (M-CSF)-differentiated primary human macrophages were first methionine depleted for 1 h and subsequently treated with HPG for up to 6 h in the presence or absence of homoharringtonine, a protein translation inhibitor. HPG accumulated in macrophages over time as measured by flow cytometry (Figure 2B). This accumulation was entirely blocked by treatment with homoharringtonine (Figure 2C), demonstrating that HPG incorporation can be used as a proxy for protein synthesis.

Next, to test whether CENCAT could reproduce the results of the original SCENITH protocol using the anti-puromycin antibody clone R4743L-E8, both assay variants were applied side by side to analyze the metabolic profiles of primary human macrophages after classical activation (lipopolysaccharide [LPS] + interferon [IFN]γ) vs. alternative activation (interleukin [IL]-4). Representative histograms of protein synthesis for the different inhibitor conditions are shown in Figure 2D for puromycin and Figure 2E for HPG. LPS activation in macrophages is associated with the upregulation of glycolysis and a disrupted tricarboxylic acid (TCA) cycle, whereas IL-4 activation is supported by enhanced mitochondrial oxidative phosphorylation (OXPHOS).^17^ As expected, classical activation resulted in a relative increase in glycolytic capacity over mitochondrial dependence. At the same time, the inverse was observed for alternative activation in both granulocyte M-CSF (GM-CSF)- (Figure 2F) and M-CSF- (Figure 2G) differentiated macrophages. This result was obtained irrespective of whether HPG or puromycin detection was used as a proxy for protein synthesis. Both GM-CSF and M-CSF macrophages were highly glucose dependent (>73%) across the different polarized states, as evidenced by both techniques (Figures S1A and S1B). The absolute values for glucose dependence were not significantly different between the two techniques (Figure S1A). These results establish CENCAT as a viable method for performing immune cell metabolic profiling.

### CENCAT reveals differential metabolic responses to inflammatory stimulation in PBMCs

**Figure 3:**
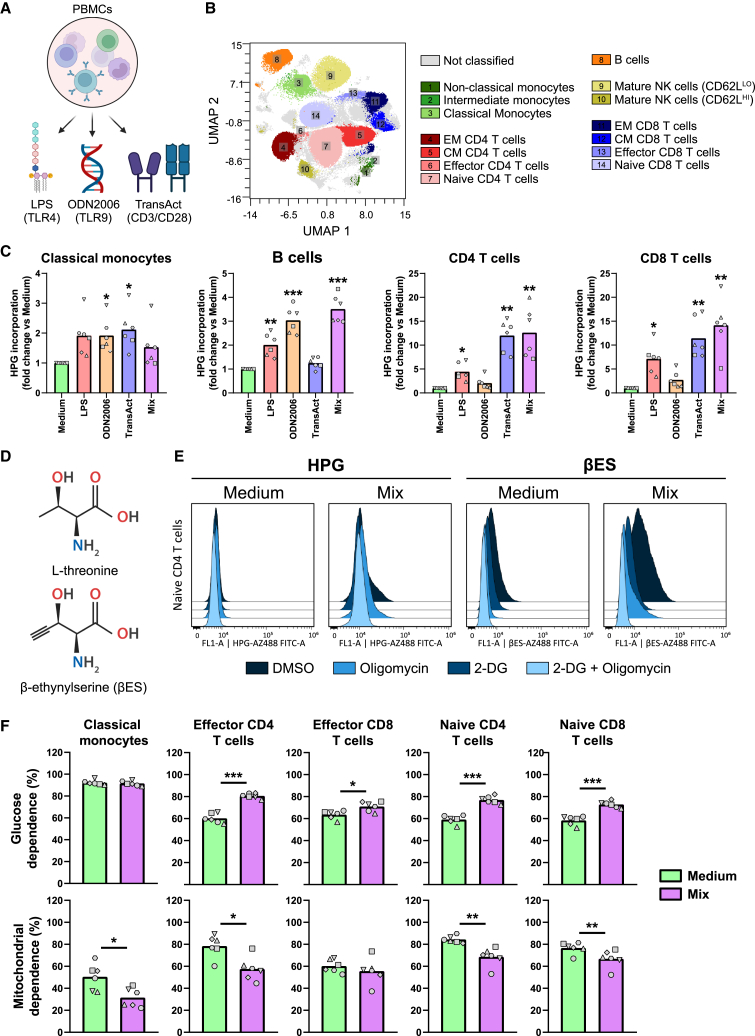
CENCAT analysis of PBMC metabolic profiles after different stimulations (A) PBMCs isolated from healthy blood donors (*n* = 6) were stimulated for 2 h with Medium control (green), LPS (red), ODN2006 (orange), TransAct (blue), or all stimuli combined (Mix, purple). (B) Representative uniform manifold approximation and projection (UMAP) plot of PBMCs stained with 9-marker flow cytometry panel. Colors and numbers indicate different cell populations. (C) Mean fold changes of HPG incorporation compared to Medium control of classical monocytes, B cells, CD4 T cells, and CD8 T cells. Significance was tested by repeated one-way ANOVA with Dunnett’s multiple comparisons test. Individual donors are displayed by different symbols. ∗*p* < 0.05, ∗∗*p* < 0.01, and ∗∗∗*p* < 0.001. (D) Structures of L-threonine and βES. (E) Representative histograms of protein synthesis for DMSO, 2-DG, oligomycin, and 2-DG + oligomycin conditions as measured using HPG or βES in naive CD4 T cells. (F) Mean glucose dependence (%) and mitochondrial dependence (%) of classical monocytes, effector CD4 T cells, effector CD8 T cells, naive CD4 T cells, and naive CD8 T cells. Significance was tested by paired t test. Individual donors are displayed by different symbols. ∗*p* < 0.05, ∗∗*p* < 0.01, and ∗∗∗*p* < 0.001. CENCAT was performed using HPG (C and E) or βES (E and F).

An essential improvement of SCENITH over other techniques, such as Seahorse XF analysis, is its ability to perform metabolic profiling of multiple cell types simultaneously in complex samples. Therefore, we next tested the applicability of HPG-based CENCAT to determine metabolic dependencies in PBMCs using a nine-marker flow cytometry panel to identify B cells, monocytes, CD4 T cells, CD8 T cells, and natural killer (NK) cells (Figures 3B and S2 for gating strategy). We aimed to investigate the metabolic profile of these cells in both their resting state and after immune cell activation. A combination of immunological stimuli was applied to achieve this, as different immune cell types are activated through distinct cell surface receptors. For instance, myeloid cells, such as monocytes, highly express the Toll-like receptor 4 (TLR4) receptor, which recognizes LPS. B cells respond strongly to DNA containing unmethylated CpG sequences via TLR9,^18^^,^^19^ whereas T cells are activated by co-activating the T cell co-receptor CD3 and its co-stimulatory receptor CD28. Therefore, to determine the effect of immune activation on metabolic dependencies, PBMCs were either left untreated (medium) or stimulated for 2 h with LPS (TLR4 ligand), ODN2006 (CpG, TLR9 ligand), TransAct (synthetic CD3/CD28 agonist), or a mixture containing all three ligands (Figure 3A).

HPG incorporation was differentially elevated by stimulation in all tested immune cells (Figure 3C). The stimuli mixture (Mix) generally yielded similar results to the most potent single stimulation for each cell type. As expected, B cells showed the highest HPG incorporation after treatment with the TLR9 ligand ODN2006, while both CD4 and CD8 T cells most strongly responded to CD3/CD28 activation by TransAct. CD14^+^/CD16^−^ classical monocytes showed a similar increase in HPG levels for all single stimulations, most likely due to cytokine production by other PBMCs. Noticeably, our results showed that HPG incorporation levels were low in several cell types. This was most apparent in untreated T cells, indicating that these cell types are relatively quiescent when inactive (Figure S3A). As a result, small fluctuations in HPG signals skew the relative metabolic dependencies in these cells, leading to high variability (Figures S3B and S3C). Additionally, oligomycin treatment occasionally increased protein translation in T cells, leading to negative values for mitochondrial dependence.

As HPG incorporation is known to be inefficient relative to methionine,^20^ we next tested βES (Figure 3D), a novel threonine-derived ncAA that reportedly has a ∼12.5-fold higher incorporation rate compared to HPG.^13^ The use of βES as a substrate for CENCAT was analyzed in PBMCs left either unstimulated (Medium) or treated with the three ligand stimulation mixture (Mix) for 2 h βES displayed improved incorporation in T cells compared to HPG, as is illustrated by the flow cytometry histograms of naive CD4 T cells in Figure 3E. Only a low HPG signal was detected in the control condition (ΔMFI DMSO vs. 2-DG + oligomycin: 937 ± 578), which was elevated after stimulation (ΔMFI: 6,547 ± 4,139). Incorporation was significantly higher in naive CD4 T cells incubated with βES (Medium control ΔMFI: 6,715 ± 3,909, *p* = 0.035 vs. HPG; stimulation Mix ΔMFI: 12,865 ± 6,629, *p* = 0.018 vs. HPG). Furthermore, unlike HPG, βES-treated cells did not display any negative metabolic dependencies (Figures 3F, S4C, and S4D).

Principal-component analysis (PCA) showed a clear group separation between samples stimulated with the stimulation mixture or medium control (Figure S4A). The top loadings of the first component (PC1) show that metabolic dependencies of classical monocytes and CD45RA^+^ T cells, comprising the effector and naive subsets, were most important for group separation along this axis (Figure S4B). Stimulation significantly decreased mitochondrial dependence in these cell types except for effector CD8 T cells (Figure 3F), indicating a metabolic switch to glycolysis reminiscent of the Warburg effect. In line with this, activated CD45RA^+^ T cell subsets displayed a significantly increased dependence on glucose, whereas glucose dependence was already high (>90%) in classical monocytes at basal conditions. This increased reliance on glucose was also observed in most CD45RA^−^ central/effector memory T cell subsets (Figure S4C); however, these cells did not display a relative glycolytic shift in response to stimulation. Compared to classical monocytes, the non-classical and intermediate subsets showed a similar high dependence on glucose at baseline (Figure S4D) but a much higher mitochondrial dependence (90%–98%). Unfortunately, these subsets could no longer be detected after stimulation. B cells and mature NK cells did not show a shift in metabolism in response to the stimulation mixture. Altogether, owing to increased incorporation and, as a result, improved resolution of protein synthesis, we propose the use of βES over HPG for metabolic profiling of complex samples containing relatively quiescent cells, such as naive T lymphocytes.

Finally, we performed a side-by-side comparison of CENCAT using βES with the current gold standard in metabolic profiling, Seahorse XF. CD14^+^ monocytes were isolated from buffy coats of healthy blood bank donors (*n* = 3, donors A/B/C) and stimulated for 2 h with LPS (100 ng/mL) or RPMI control, after which they were analyzed by either CENCAT or Seahorse XF analysis (Figure S5A). For Seahorse, monocyte glycolytic capacity was determined after sequential injection of glucose and oligomycin (Figure S5B). Both techniques demonstrate a clear increase in the glycolytic capacity of monocytes in response to LPS (Figure S5C), although relative fold changes in glycolytic capacity between samples could differ depending on the method used. Injection of βES did not affect monocyte metabolism as determined by Seahorse XF analysis (Figure S5D), demonstrating that βES treatment by itself does not directly modulate cellular behavior.

### Metabolic profiling of murine tissue-resident immune cell populations

We next tested the potential of CENCAT with βES to determine the metabolic profiles of tissue-resident immune cell populations in mice. Immune cells from epididymal white adipose tissue (eWAT), kidneys, liver, lungs, peritoneal exudate cells (PECs), and spleen were isolated from wild-type C57BL/6J mice and subjected to CENCAT with βES. A 12-marker flow cytometry was used to identify conventional dendritic cell subsets (cDC1 and cDC2), tissue-specific macrophage populations, monocytes, B cells, neutrophils, eosinophils, and CD4 and CD8 T cells. The complete gating strategies are depicted in Figure S2.

**Figure 4:**
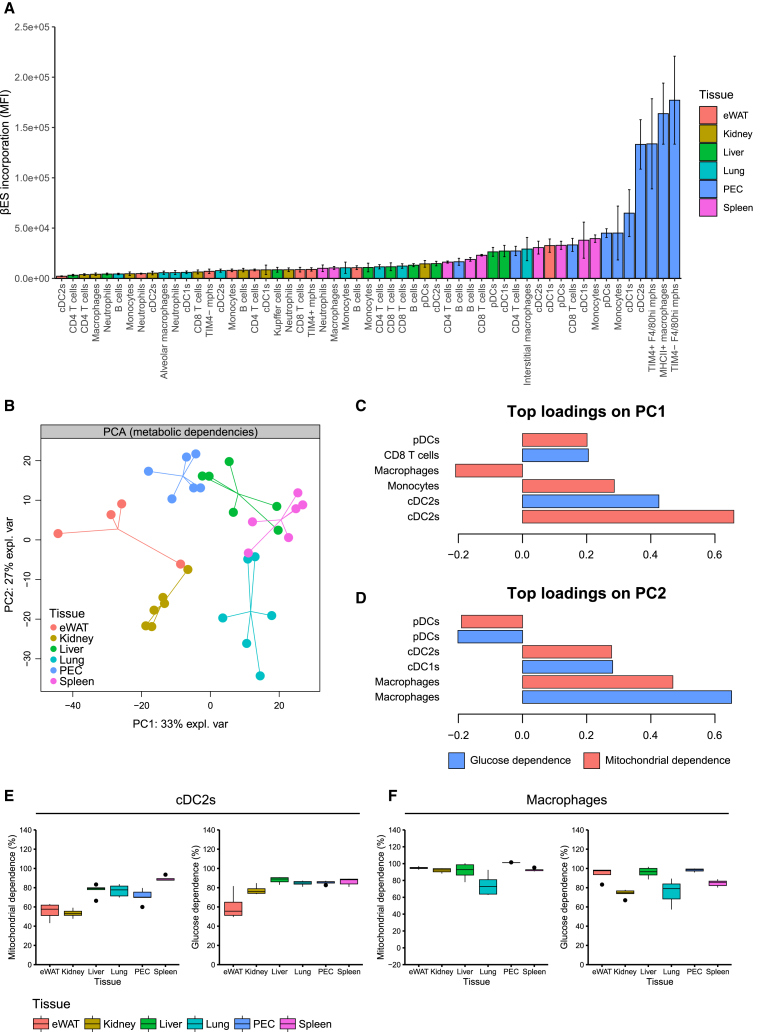
CENCAT analysis of murine tissue-resident immune cell populations The following tissues were isolated from male C57BL/6J mice and subjected to CENCAT analysis: eWAT (red, *n* = 4), kidney (yellow, *n* = 6), liver (green, *n* = 6), lung (cyan, *n* = 6), PECs (blue, *n* = 6), and spleen (pink, *n* = 6). (A) βES incorporation (MFI) of immune cell populations from all six tissues. Data are displayed as means ± SD. (B) PCA score plot based on metabolic dependencies of immune cell populations in all six tissues. (C and D) Top loadings on PC1 (C) and PC2 (D) of the PCA score plot. Measures of glucose dependence are represented by blue bars and mitochondrial dependence by red bars. (E and F) Boxplots of mitochondrial and glucose dependence (%) of cDC2s (E) and macrophages (F) from all six tissues.

βES incorporation levels were variable across tissues and cell types (Figure 4A). In general, tissue-resident immune cells in PEC and spleen samples showed the highest βES fluorescence intensities, while βES accumulation was relatively low in kidney immune cells. Within tissues, DCs and macrophages often displayed the highest βES fluorescence intensities. βES incorporation and metabolic profiles could not be assessed in eosinophils, as these cells acquired a very high non-specific background signal after the fluorescent click reaction, even in the absence of βES (data not shown).

To visualize potential differences in immune cell metabolic profiles across tissues, glucose and mitochondrial dependencies of shared immune populations were analyzed by PCA. Samples derived from the same tissue clustered together, and clear group separations were observable between different tissues (Figure 4B). The top loadings of the first and second components (PC1 and PC2), which respectively explain 33% and 27% of data variance, show that metabolic dependencies of myeloid cells were most important for projection along the axes, in particular cDC2s (Figure 4C) and macrophages (Figure 4D), indicating that these cells are the most metabolically variable across different tissues. cDC2s from eWAT and kidney showed low levels of mitochondrial and glucose dependence compared to the other tissues, while spleen cDC2s were highly reliant on glucose and mitochondria (Figure 4E).

Interstitial macrophages, Kupffer cells, and adipose tissue macrophages were similarly highly dependent on glucose and oxidative metabolism (Figures S6A and S6B). Within PECs, a subdivision in mitochondrial and glucose metabolism could be observed between major histocompatibility complex (MHC) class II+ monocyte-derived immature macrophages and F4/80^HI^ mature tissue-resident macrophages, which is in line with a recent study,^21^ which employed CENCAT with HPG. Neutrophils were the most glycolytic of all investigated cell types, particularly in adipose tissue, whereas monocytes, pDCs, cDC1s, B cells, and T cells primarily depended on oxidative metabolism (Figure S6C). Additionally, neutrophils and monocytes were highly reliant on glucose across all tissues, while this was more variable for pDCs, B cells, and T cells (Figure S6D).

## Discussion

Here, we established CENCAT as a valid approach for conducting extensive metabolic profiling of multiple cell types simultaneously. Our findings demonstrate that ncAA incorporation can serve as an alternative to puromycin immunostaining as a proxy for protein synthesis. Among the tested ncAAs, the novel threonine analog βES emerges as the preferred choice over HPG, owing to its high incorporation efficiency in metabolically inactive cells. We have shown that CENCAT is applicable for studying immune cells in PBMCs and tissue-resident immune cells from murine tissues. This underscores its significance as a potent metabolic profiling tool in the immunologist’s arsenal.

The use of ncAAs over puromycin as a substrate to measure protein synthesis has several advantages. Firstly, puromycin is a toxic antibiotic often used as a selection agent for eukaryotic cells. Puromycin inhibits protein translation, leading to the formation of prematurely terminated truncated polypeptides.^22^ This apparent toxicity limits the maximal substrate incorporation time and could lead to unwanted effects. ncAAs, on the other hand, are incorporated into full-length proteins and are, therefore, reported to be non-toxic.^12^ Secondly, puromycin labeling was found to be inappropriate for accurately measuring overall protein synthesis rates during energy-starved conditions compared to the methionine analog AHA, particularly in response to glucose starvation and 2-DG treatment.^9^ This could explain differences in glucose dependence measurements between both technologies.

Our CENCAT approach using HPG, similar to the original SCENITH assay using puromycin, showed that LPS/IFNγ-primed classical activation of monocyte-derived macrophages resulted in a relative increase in glycolytic capacity, while IL-4-induced alternatively activated macrophages displayed enhanced mitochondrial dependence. These results align with previous work in murine bone-marrow-derived macrophages^23^^,^^24^ and a recent study on Seahorse analysis of human monocyte-derived macrophage activation.^25^ A limitation of CENCAT with HPG over the original protocol is the requirement for methionine depletion before treatment, which requires specialized culture media and extra washing steps. Additionally, we observed very low levels of HPG incorporation in T cells and NK cells, indicating that these cells are metabolically quiescent under basal conditions, which is in accordance with previous research,^23^^,^^24^ yet makes it more difficult to accurately determine their metabolic dependencies. This was particularly evident for oligomycin treatment, which, in some settings, increased HPG incorporation compared to DMSO control, possibly due to metabolic adaptation through increased glycolytic flux.^26^ This phenomenon was also observed by Vogel et al. using puromycin, which they termed MITA (mitochondrial inhibition translation activation).^27^ The occurrence of MITA could potentially lead to an underestimation of mitochondrial dependence.

As an alternative approach, we tested the ncAA βES, a threonine analog with a relatively efficient incorporation rate of ∼1:40 (βES:threonine) under native culture conditions.^13^ Our results showed that βES is indeed more efficiently incorporated in both naive and activated T cells without depleting competing amino acids. Furthermore, we did not observe MITA in any tested cell types using βES, which could explain differences in mitochondrial dependence measurements compared to HPG. Application of CENCAT using βES in PBMCs replicated previously published metabolic signatures of immune cells. Upon activation, classical monocytes reportedly switch from a mitochondrial-dependent to a glycolytic profile,^28^ which could be reproduced by CENCAT. Furthermore, CD16^+^/CD14^−^ non-classical monocytes displayed higher levels of mitochondrial dependence compared to classical CD16^−^/CD14^+^ monocytes under basal conditions, which is congruent with previous studies reporting increased mitochondrial activity and transcription of genes involved in mitochondrial respiration in non-classical monocytes.^29^^,^^30^ Finally, activation of T cells increased their glycolytic capacity and/or glucose dependence as expected,^31^ particularly in CD45RA^+^ naive and effector subsets.

CENCAT results with βES were corroborated by Seahorse XF analysis in LPS-stimulated monocytes, although some differences in effect size could be observed at the individual level. For instance, the basal glycolytic capacity of monocytes from donor A appeared higher in CENCAT results compared to Seahorse. However, both parameters should be interpreted differently: glycolytic capacity as assessed by CENCAT indicates the relative contribution of glycolysis to protein synthesis, while this value for Seahorse indicates the absolute level of glycolysis as reflected by the ECAR. Theoretically, the MFI of βES incorporation should also indicate the absolute levels of glycolysis; however, this value should be interpreted with care, as it reflects nascent synthesis of intracellular but not secreted proteins. This could potentially be amended by the addition of an inhibitor of protein secretion during the CENCAT assay (e.g., brefeldin A).

Although studying circulating immune cell populations can yield valuable insights, it is important to acknowledge that they can differ significantly from their tissue-resident counterparts, which possess unique functions and metabolic profiles driven by microenvironmental imprinting. Until now, there has been a lack of suitable technologies to measure the metabolism of tissue-resident immune cells due to low cell availability and high sample complexity. By employing CENCAT on murine tissues, we could separate samples according to their tissue residency. Our results showed that the degree of βES incorporation was variable between tissues, with high levels detected in spleen and peritoneal immune cells, while the βES signal was generally low in kidney samples. This is potentially related to the purity of the sample, as cells from the peritoneum and spleen are almost solely of leukocytic origin, whereas kidney samples still contain other cell types and biological material, which could interfere with βES uptake, even after CD45^+^ magnetic cell sorting. Metabolic dependencies of cDC2s and macrophage subsets were the main drivers of the separation between tissues. We further observed that tissue-resident cDC1s were generally more mitochondrially dependent than their cDC2 counterparts. Consistent with this finding, *in*-*vitro*-generated cDC1s were reported to have an increased mitochondrial content and membrane potential compared to cDC2s.^32^ Among all tissues, neutrophils were found to be the most glycolytic of all studied cell types, which is congruent with current literature.^33^^,^^34^ Within macrophage populations, kidney macrophages incorporated very low levels of βES, in line with their reported quiescent state during homeostasis.^35^ Furthermore, our results corroborate previous reports that peritoneal macrophages are highly mitochondrially dependent.^36^^,^^37^ However, some of our results contradict a ranking of murine tissue macrophages based on their relative expression of OXPHOS-related genes in single-cell RNA sequencing datasets by Wculek and colleagues.^38^ For example, they showed alveolar macrophages among the highest-ranking subsets, while we found these cells to be relatively glycolytic. This indicates that OXPHOS gene signatures may not directly translate to a functional preference of mitochondrial over glycolytic metabolism.

In summary, CENCAT is a promising technique for performing metabolic profiling of samples of varying complexity, spanning isolated cell types to *ex vivo* murine tissues. Our adaptation retains the benefits of the original protocol, including its independence of specialized equipment except for a flow cytometer, low cell number requirement, and non-necessity for cell purification. Compared to the original protocol, the use of ncAAs as a proxy for metabolic activity is preferable over puromycin due to their lack of toxicity. Based on our outcomes, βES is the superior ncAA for profiling complex samples containing cell types with variable metabolic activity. While tested here with a focus on immune cells, CENCAT is not limited by cell type or tissue and can also be applied in other research areas beyond immunometabolism.

### Limitations of the study

While the use of flow cytometry allows for single-cell resolution, CENCAT is currently not a true single-cell approach. Due to the requirement for fixation and permeabilization for CuAAC, the metabolic inhibitors currently need to be applied in parallel using separate samples, allowing for the analysis of cell subpopulations but not individual cells. Single-cell resolution could be achieved in the future by using live-cell click-compatible ncAAs and cell-permeable fluorescent probes.

## Resource availability

### Lead contact

Further information and requests for resources and reagents should be directed to the lead contact, Rinke Stienstra (rinke.stienstra@wur.nl).

### Materials availability

βES-HCl is available from Kimberly Bonger’s lab upon request.

### Data and code availability

• All data reported in this paper are available to download from Mendeley Data (https://doi.org/10.17632/k2snk9z8jc.1) or from the lead contact upon request.
• This paper does not report any original code.
• Any additional information required to reanalyze the data reported in this paper is available from the lead contact upon request.

## Acknowledgments

We thank 10.13039/501100001826ZonMw for funding R.S. and H.J.P.v.d.Z. (“The right timing to prevent type 2 Diabetes (TIMED),” project number 459001021). Figure 1 and the graphical abstract were created using BioRender (www.biorender.com).

## Author contributions

F.V. conceived the study, performed experiments, and wrote the manuscript. H.J.P.v.d.Z., L.S., B.N., and J.I.P.v.H. performed experiments and reviewed the manuscript. F.V. and H.J.P.v.d.Z. performed the data analyses. B.J.I., K.M.B., and J.V.d.B. provided crucial reagents. S.K. and R.S. supervised the study and reviewed the manuscript.

## Declaration of interests

The authors declare no competing interests.

## STAR★Methods

### Key resources table

**Table:** 

REAGENT or RESOURCE	SOURCE	IDENTIFIER
**Antibodies**		

Anti-human CD4-BV605 (clone RPA-T4)	Biolegend	Cat#300556
Anti-human CD8-BV650 (clone RPA-T8)	Biolegend	Cat#301042
Anti-human CD14-APC (clone 63D3)	Biolegend	Cat#367118
Anti-human CD16-PerCP-Cy5.5 (clone 3G8)	Biolegend	Cat#302028
Anti-human CD19-PE-Dazzle594 (clone HIB19)	Biolegend	Cat#302252
Anti-human CD45RA-BV785 (clone HI100)	Biolegend	Cat#304140
Anti-human CD62L-BV421 (clone DREG-56)	Biolegend	Cat#304828
Anti-human HLA-DR-PE-Cy7 (clone L243)	Biolegend	Cat#307616
Anti-human CD56-PE (clone NCAM16.2)	BD Biosciences	Cat#345812
Anti-human CD14-BUV395 (clone MϕP-9)	BD Biosciences	Cat#563561
Anti-human HLA-DR-BUV661 (clone G46-6)	BD Biosciences	Cat#612980
Anti-mouse/human CD45R/B220-BV510 (clone RA3-6B2)	Biolegend	Cat#103248
Anti-mouse CD4-FITC (clone GK1.5)	Biolegend	Cat#100405
Anti-mouse CD8a-PE-Cy7 (clone 53–6.7)	Biolegend	Cat#100721
Anti-mouse/human CD11b-BV650 (clone M1/70)	Biolegend	Cat#101259
Anti-mouse CD11c-BV605 (clone N418)	Biolegend	Cat#117333
Anti-mouse CD45-PerCP-Cy5.5 (clone 30-F11)	Biolegend	Cat#103131
Anti-mouse CD64-APC (clone X54-5/7.1)	Biolegend	Cat#139305
Anti-mouse F4/80-FITC (clone BM8)	Biolegend	Cat#123108
Anti-mouse Ly-6C-Alexa Fluor 700 (clone HK1.4)	Biolegend	Cat#128024
Anti-mouse I-A/I-E (MHCII)-BV785 (clone M5/114.15.2)	Biolegend	Cat#107645
Anti-mouse CD170 (Siglec-F)-PE-Dazzle594 (clone S17007L)	Biolegend	Cat#155529
Anti-mouse TIM4-PE (clone RMT4-54)	Biolegend	Cat#130005
Anti-mouse CLEC2-FITC (clone 17D9)	Bio-Rad	Cat#MCA5700

**Chemicals, peptides, and recombinant proteins**		

β-ethynylserine-HCl (βES)	Bonger Lab, Radboud University^13^	NA
L-homopropargylglycine (HPG)	Vector Laboratories	Cat#CCT-1067
THPTA	Vector Laboratories	Cat#CCT-1010
AZDye 405 Azide Plus	Vector Laboratories	Cat#CCT-1474
AZDye 488 Azide Plus	Vector Laboratories	Cat#CCT-1475
Lipopolysaccharide from *Escherichia coli* O55:B5	Merck	Cat#L6529
Oligomycin A	Merck	Cat#75351
2-deoxy-D-glucose (2-DG)	Merck	Cat#D8375
L-cystine dihydrochloride	Merck	Cat#C6727
RPMI 1640 medium (with sodium bicarbonate, without methionine, cystine and L-glutamine)	Merck	Cat#R7513
Homoharringtonine	Merck	Cat#SML1091
Fetal Calf Serum (FCS)	Biowest	Cat#S1300
RPMI 1640 medium (with sodium bicarbonate, without L-glutamine and HEPES)	Thermo Fisher	Cat#21870076
GlutaMAX™	Thermo Fisher	Cat#35050061
Fetal Bovine Serum, dialyzed, US origin, One Shot™ format	Thermo Fisher	Cat#A3382001
Recombinant Human Interferon γ (IFNγ)	Peprotech	Cat#300-02
Recombinant Human Interleukin 4 (IL-4)	Peprotech	Cat#200-04
ODN2006	Hycult	Cat#HC4039
Puromycin	Invivogen	Cat#ant-pr-1
T cell TransAct™, human	Miltenyi Biotec	Cat#130-128-758
Penicillin-Streptomycin Solution	Corning	Cat#30-002-CI
Zombie NIR Fixable Viability Kit	Biolegend	Cat#423106
Zombie Aqua Fixable Viability Kit	Biolegend	Cat#423102
Human TruStain FcX	Biolegend	Cat#422302
Mouse TruStain FcX	Biolegend	Cat#101320
True-Stain Monocyte Blocker	Biolegend	Cat#426103
CF700 Succinimidyl Ester	Biotium	Cat#96067
CF750 Succinimidyl Ester	Biotium	Cat#92142
eBioscience™ Permeabilization Buffer (10X)	Invitrogen	Cat#00-8333-56
Brilliant Stain Buffer Plus	BD Biosciences	Cat#566385
Ficoll Paque Plus	Merck	Cat#GE17-1440-03
Recombinant Human M-CSF	Miltenyi Biotec	Cat#130-096-489
Recombinant Human GM-CSF	Miltenyi Biotec	Cat#130-093-864
Collagenase type II from *Clostridium histolyticum*	Merck	Cat#C6885
Collagenase V	Merck	Cat#C9263
Dispase II	Merck	Cat#D4693
Collagenase D *from Clostridium histolyticum*	Merck	Cat#11088858001
DNase I	Merck	Cat#4536282001
Seahorse XF RPMI Medium pH 7.4	Agilent Technologies	Cat#103576-100

**Critical commercial assays**		

MojoSort Human CD14 Nanobeads	Biolegend	Cat#480094
LS columns	Miltenyi Biotec	Cat#130-042-401
CD45 MicroBeads, mouse	Miltenyi Biotec	Cat#130-052-301
Seahorse XFe96/XF Pro FluxPak	Agilent Technologies	Cat#103792-100
Leucosep™ tubes	Greiner Bio-One	Cat#227288

**Experimental models: organisms/strains**		

Mouse: C57BL/6J (B6)	The Jackson Laboratory	RRID:IMSR_JAX:000664

**Software and algorithms**		

FlowJo software version 10.8.1	BD Biosciences	https://www.bdbiosciences.com/en-eu/products/software/flowjo-v10-software
OMIQ	Dotmatics	https://www.omiq.ai
R version 4.2.2	R Core Team	https://www.r-project.org/
GraphPad Prism software version 8.01	Dotmatics	https://www.graphpad.com/features
ggplot2 version 3.4.22	Wickham et al.^20^	https://cran.r-project.org/web/packages/ggplot2/index.html
cowplot version 1.1.1	Wilke et al.^21^	https://cran.r-project.org/web/packages/cowplot/index.html
ggh4x 0.2.4	van den Brand et al.^22^	https://cran.rstudio.com/web/packages/ggh4x/index.html

### Experimental model and study participant details

#### Human participants

PBMCs were isolated from healthy, female volunteers aged between 22 and 30. Ethical approval was obtained from the CMO Arnhem-Nijmegen, the Netherlands (NL32357.091.10). Human primary monocytes were isolated from buffy coats of healthy blood bank donors from which information regarding age, sex or gender identity could not be provided due to anonymity of the donors. All blood samples were collected after acquiring written informed consent as per the norms of the International Declaration of Helsinki.

#### Animals

All experiments followed the Guide for the Care and Use of Laboratory Animals of the Institute for Laboratory Animal Research and were approved by the Central Authority for Scientific Procedures on Animals (CCD, AVD10400202115283) and the Institutional Animal Care and Use Committee of Wageningen University. Tissues were collected from mice as part of experimental protocols 2021.W-0016.008 and 2021.W-0016.007. Naive, 12-16-week-old, male wild-type C57BL/6J mice were used in this study. The mice were co-housed with 2–3 mice per cage in a temperature-controlled room with a 12h light-dark cycle and *ad libitum* access to food and tap water.

### Method details

#### Peripheral blood mononuclear cell (PBMC) isolation and stimulation

EDTA blood tubes were pooled and diluted 1:1 in PBS, after which PBMCs were isolated by Ficoll Paque Plus (Merck) gradient centrifugation in Leucosep tubes (Greiner Bio-One). PBMC layers were collected in a 50 mL tube and washed thrice with PBS before counting using a hemocytometer. PBMCs were resuspended at a density of 10×10^6^ cells/mL in RPMI 1640 medium supplemented with 10% FCS, GlutaMAX, and P/S and cultured in FACS tubes at 37°C/5%CO_2_ while shaking (100 RPM). To activate PBMCs, cells were stimulated with LPS (100 ng/mL), ODN 2006 (1 μM), TransAct (1:100), or a mixture of all three stimuli for 2 h in total.

#### Human monocyte isolation and macrophage differentiation

Human primary monocytes were isolated from buffy coats of healthy blood bank donors through magnetic-activated cell sorting (MACS). After PBMC isolation as described above, CD14^+^ monocytes were magnetically labeled using MojoSort Human CD14 Nanobeads (Biolegend) and subsequently separated on LS columns using a QuadroMACS Separator (Miltenyi Biotec BV). Isolated monocytes were counted using a Vi-CELL XR Cell Analyzer (Beckman Coulter), resuspended at a density of 1×10^6^ cells/mL in RPMI 1640 medium supplemented with 10% FCS, GlutaMAX and P/S, and seeded in T75 flasks using 10 mL per flask (10×10^6^ cells). Monocytes were cultured for six days at 37°C/5%CO_2_ in the presence of either M-CSF (50 ng/mL; Miltenyi) or GM-CSF (5 ng/mL; Miltenyi) for macrophage differentiation. At day 3 of differentiation, 5 mL of fresh medium containing M-CSF or GM-CSF was added to each flask.

After differentiation, macrophages were harvested by trypsinization and seeded in 24-well plates at a density of 3×10^5^ cells/well (GM-CSF) or 4×10^5^ cells/well (M-CSF). Macrophages were subsequently left untreated, classically activated by a combination of LPS (100 ng/mL) and IFNγ (10 ng/mL), or alternatively activated using IL-4 (20 ng/mL) for 24 h.

#### Mice experiments

All experiments followed the Guide for the Care and Use of Laboratory Animals of the Institute for Laboratory Animal Research and were approved by the Central Authority for Scientific Procedures on Animals (CCD, AVD10400202115283) and the Institutional Animal Care and Use Committee of Wageningen University. Tissues were collected from mice as part of experimental protocols 2021.W-0016.008 and 2021.W-0016.007. Naive, 12-16-week-old, male wild-type C57BL/6J mice were sacrificed by cervical dislocation, and epidydimal white adipose tissue (eWAT) fat pads, kidneys, liver, lungs, and spleen were collected in RPMI for tissue-resident immune cell isolation. Before collecting tissues, the peritoneal cavity was washed with 10 mL ice-cold PBS supplemented with two mM EDTA to isolate peritoneal exudate cells (PECs). Tissue-resident immune cells were isolated as described previously^39^^,^^40^ with minor adaptations. Briefly, eWAT, kidneys, liver, and lungs were cut into small pieces using razors, and the spleen was mechanically disrupted using a syringe plunger. eWAT fat pads were digested in 5 mL digestion buffer containing 1 mg/mL collagenase type II from *Clostridium histolyticum* (Merck) in Krebs buffer supplemented with 100 mM HEPES, 20 mg/mL BSA and 6 mM D-Glucose for 45 min at 37°C/5%CO_2_ while shaking (100 RPM). Digestion was stopped by adding 5 mL wash buffer (PBS supplemented with 1% FCS and 2.5 mM EDTA), and the solution was filtered through 250 μm Nitex filters (Sefar). Infranatant containing SVF was collected, and erythrocytes were lysed using ice-cold erythrocyte lysis buffer (in-house, 0.15 M NH_4_Cl, 1 mM KHCO_3_, 0.1 mM Na_2_EDTA). Cells were finally filtered through 40 μm cell strainers (PluriSelect).

Kidneys and lungs were digested in 5 mL digestion buffer containing 1 mg/mL collagenase V, 1 mg/mL dispase II, and 30 U/mL DNase I in RPMI for 30 min at 37°C/5%CO_2_ while shaking (100 RPM). Digestion was stopped by adding 5 mL wash buffer, and the digest was filtered through 100 μm cell strainers (PluriSelect). After erythrocyte lysis and filtering through 40 μm cell strainers, leukocytes were purified using magnetic-assisted cell sorting (MACS) using CD45 microbeads (35 μL per sample, Miltenyi Biotec) according to manufacturer’s instructions.

The liver was digested in 5 mL RPMI supplemented with 1 mg/mL collagenase V, 1 mg/mL dispase II, 1 mg/mL collagenase D, and 30 U/mL DNase I for 25 min at 37°C/5%CO_2_ while shaking (100 RPM). Digest was filtered through 100 μm cell strainers and washed twice with 40 mL wash buffer (300 RCF, 5 min, 4°C). After erythrocyte lysis and filtering through 40 μm cell strainers, leukocytes were isolated using CD45 MACS, similar to kidneys and lungs.

The spleen was digested in 5 mL digestion buffer containing 1 mg/mL collagenase D and 30 U/mL DNase I in RPMI for 30 min at 37°C/5%CO_2_ while shaking (100 RPM). Digest was filtered through 100 μm filters, incubated with erythrocyte lysis buffer, and filtered again through 40 μm filters.

All isolated and filtered cells were counted using a hemocytometer. eWAT, kidney, and peritoneal leukocytes were split over four wells, 2.5–5x10^5^ liver and lung leukocytes/well, and 1 × 10^6^ splenocytes/well were plated in 96-well U-bottom plates for CENCAT.

#### Seahorse XF analysis

CD14^+^ monocytes were seeded in quintuple in XF96 microplates (250,000 cells/well; Agilent Technologies) in RPMI 1640 medium supplemented with GlutaMAX and 10% FCS. Cells were either left unstimulated or treated with LPS (100 ng/mL) for 2 h in total at 37°C/5%CO_2_. After 60 min, RPMI medium was replaced by Seahorse XF RPMI Medium pH 7.4 (Agilent Technologies) without glucose supplemented with GlutaMAX and incubated for another 60 min in a CO_2_-free incubator at 37°C to perform the glycostress test using the following injections: (A) D-glucose (11 mM), (B) oligomycin (1 μM). Oxygen consumption rate (OCR) and extracellular acidification rate (ECAR) were measured using a Seahorse XF96 Extracellular Flux Analyzer (Agilent Technologies). Glycolytic capacity was determined as the difference between oligomycin-induced ECAR and basal ECAR.

In a second experiment, βES was injected to monocytes (500 μM final concentration) in Seahorse XF RPMI Medium pH 7.4 supplemented with 11 mM glucose and GlutaMAX to determine the direct effect of βES on ECAR/OCR.

##### CENCAT

###### Methionine depletion for experiments with HPG

Cells (human PBMCs, human primary macrophages) incubated with HPG were first cultured for 30–45 min at 37°C/5%CO_2_ in methionine-free RPMI 1640 supplemented with 65 mg/L L-cystine dihydrochloride, 10% dialyzed FCS, GlutaMAX and P/S to deplete intracellular methionine levels. Any ongoing stimulation (e.g., LPS, ODN 2006, TransAct) was refreshed in this medium.

###### Metabolic inhibitor and ncAA incubation

βES was synthesized as described earlier^13^ and dissolved in an equimolar NaOH solution to neutralize the pH. Cells were pre-incubated with metabolic inhibitors (10× work stocks in complete RPMI) for 15 min to ensure complete shutdown of glucose and/or mitochondrial metabolism. Methionine-free RPMI was used for HPG experiments. The following four treatments were applied in technical duplicate human PBMCs, human primary macrophages) or uniplo (mouse tissue-resident immune cells) per experimental condition: DMSO (control), 2-deoxyglucose (2-DG; 100 mM), oligomycin (1 μM), and a combination of 2-DG and oligomycin. All treatments were corrected for DMSO and H_2_O content. Cells were subsequently treated with HPG (100 μM) or βES (500 μM) and incubated for 30 min at 37°C/5%CO_2_. After ncAA incorporation, macrophage samples were washed 1× with PBS, harvested by scraping, and transferred to a V-bottom 96-well plate. PBMC samples and mouse tissue-resident immune cells were transferred to a V-bottom 96-well plate and pelleted through centrifugation at 500RCF for 3 min, after which the supernatant was discarded by firmly flicking the plate once. Cells were then washed 1× with PBS. For samples stained according to the original SCENITH protocol, puromycin (end concentration 10 μg/mL) was added instead of ncAAs.

HPG was added directly after methionine depletion with or without homoharringtonine (20 μg/mL) or DMSO control for kinetic experiments. Macrophages were harvested at T = 0, 0.5, 1, 2, 4, and 6 h post-treatment.

###### Fluorescent labeling of nascent proteins and flow cytometry

Human cells were pelleted by centrifugation and incubated with Zombie Aqua Fixable Viability Dye (1:1000 in PBS) for 5–10 min at RT in the dark. Cells were then pelleted by centrifugation and fixed in 2% formaldehyde for 15 min at RT. After fixation, cells were pelleted and washed 1× with PBS before permeabilization with 0.1% saponin in PBS/1%BSA or 1X permeabilization buffer (Invitrogen) for 15 min at RT. Next, cells were washed 1× with PBS and barcoded with amine-binding dyes ((CF700-SE and CF750-SE), 1 mM stocks) for 5 min at RT. Barcoding dyes were applied in four combinations of two dilutions (1:1000 and 1:100,000), allowing for pooling the four treatment conditions per replicate. Cells were then washed 1× with PBS, and barcoded samples were pooled in Click buffer (100 mM Tris-HCl, pH 7.4). ncAAs were labeled through copper(I)-catalyzed azide-alkyne cycloaddition (CuAAC) using the following reaction mix in Click Buffer: 0.5 mM Cu(II)SO_4_, 10 mM sodium ascorbate, 2 mM THPTA and 0.5 μM AF488 Azide Plus. Freshly prepared sodium ascorbate solution (100 mM stock) and AZDye 488 Azide Plus were added to the mixture just before addition to the samples. Click reaction was performed for 30 min at RT, after which the cells were washed 2× with FACS buffer (PBS/1%BSA +2 mM EDTA). For samples stained according to the original SCENITH protocol, Alexa Fluor 488-conjugated anti-puromycin antibody diluted 1:100 in FACS buffer was added instead of the click reaction mix. PBMC samples were then blocked with Human TruStain FcX Fc Receptor Blocking Solution (1:100) before staining with fluorescent antibodies for 15 min at RT in the presence of Brilliant Stain Buffer. Antibody panel: CD4-BV605, CD8-BV650, CD14-APC, CD56-PE, CD16-PerCP-Cy5.5, CD19-PE-Dazzle594, CD45RA-BV785, CD62L-BV405 and HLA-DR-PE-Cy7. Cells were washed 1× with FACS buffer after staining and acquired on a CytoFLEX S cytometer (Beckman Coulter).

Mouse cells were processed similarly with minor adjustments. Mouse cells were not barcoded, 0.5 μM AF405 Azide Plus was used for CuAAC, and mouse tissue-resident immune cells were stained with a fluorescent antibody cocktail in the presence of Brilliant Stain Buffer and TrueStain Monocyte Blocker. Antibody panel: B220-BV510, CD4-FITC, CD8a-PE-Cy7, CD11b-BV650, CD11c-BV605, CD45-PerCP-Cy5.5, CD64-APC, F4/80-FITC, Ly6C-Alexa Fluor 700, MHCII-BV785, Siglec-F-PE-Dazzle594, TIM4-PE and CLEC2-FITC. Cells were washed 1× with FACS buffer after staining and acquired on a CytoFLEX S cytometer.

Human CD14^+^ monocytes were stained with 1 μM AF405 Azide Plus for CuAAC, followed by antibody staining for anti-human CD14-BUV395 and HLA-DR-BUV661 and acquired on a CytoFLEX LX cytometer (Beckman Coulter).

### Quantification and statistical analysis

CENCAT parameters were calculated as described previously by Argüello et al.^6^*:*

$$Mitochondrial\;dependence\;\left(\%\right)=\frac{MFI\left(DMSO\right)-MFI\left(Oligomycin\right)}{MFI\left(Basal\right)-MFI\left(2DG+Oligomycin\right)}\times 100$$

$$Glucose\;dependence\;\left(\%\right)=\frac{MFI\left(Basal\right)-MFI\left(2DG\right)}{MFI\left(Basal\right)-MFI\left(2DG+Oligomycin\right)}\times 100$$

Glycolyticcapacity(%)=100−Mitochondrialdependence

FAO/AAOcapacity(%)=100−Glucosedependence

Human macrophage and murine tissue flow cytometry data were analyzed using FlowJo software version 10.8.1 (Becton Dickinson). Human PBMC flow cytometry data were analyzed using OMIQ software from Dotmatics.

Principal component analysis (PCA) was performed in R version 4.2.2 using the mixOmics package version 6.23.4.^41^ R plots were made using the following packages: ggplot2 version 3.4.22,^42^ cowplot version 1.1.1,^43^ and ggh4x 0.2.4.^44^ Statistical significance was tested by Repeated One-Way ANOVA with Dunnett’s multiple comparisons test or two-Way ANOVA with Sidak correction for multiple testing using GraphPad Prism software version 8.01.

## Supplemental information

Document S1. Figures S1–S6

Document S2. Article plus supplemental information
