# Supplementary material for: CENCAT enables immunometabolic profiling by measuring protein synthesis via bioorthogonal noncanonical amino acid tagging
Source: Cell Rep Methods. 2024 Oct 21;4(10):100883. doi: 10.1016/j.crmeth.2024.100883 (PMC11573747; doi:10.1016/j.crmeth.2024.100883)
Supplement: Document S1. Figures S1–S6 [file mmc1.pdf]

Cell Reports Methods, Volume *4*

Supplemental information

CENCAT enables immunometabolic profiling by measuring protein synthesis via bioorthogonal noncanonical amino acid tagging

Frank Vrieling, Hendrik J.P. van der Zande, Britta Naus, Lisa Smeehuijzen, Julia I.P. van Heck, Bob J. Ignacio, Kimberly M. Bonger, Jan Van den Bossche, Sander Kersten, and Rinke Stienstra

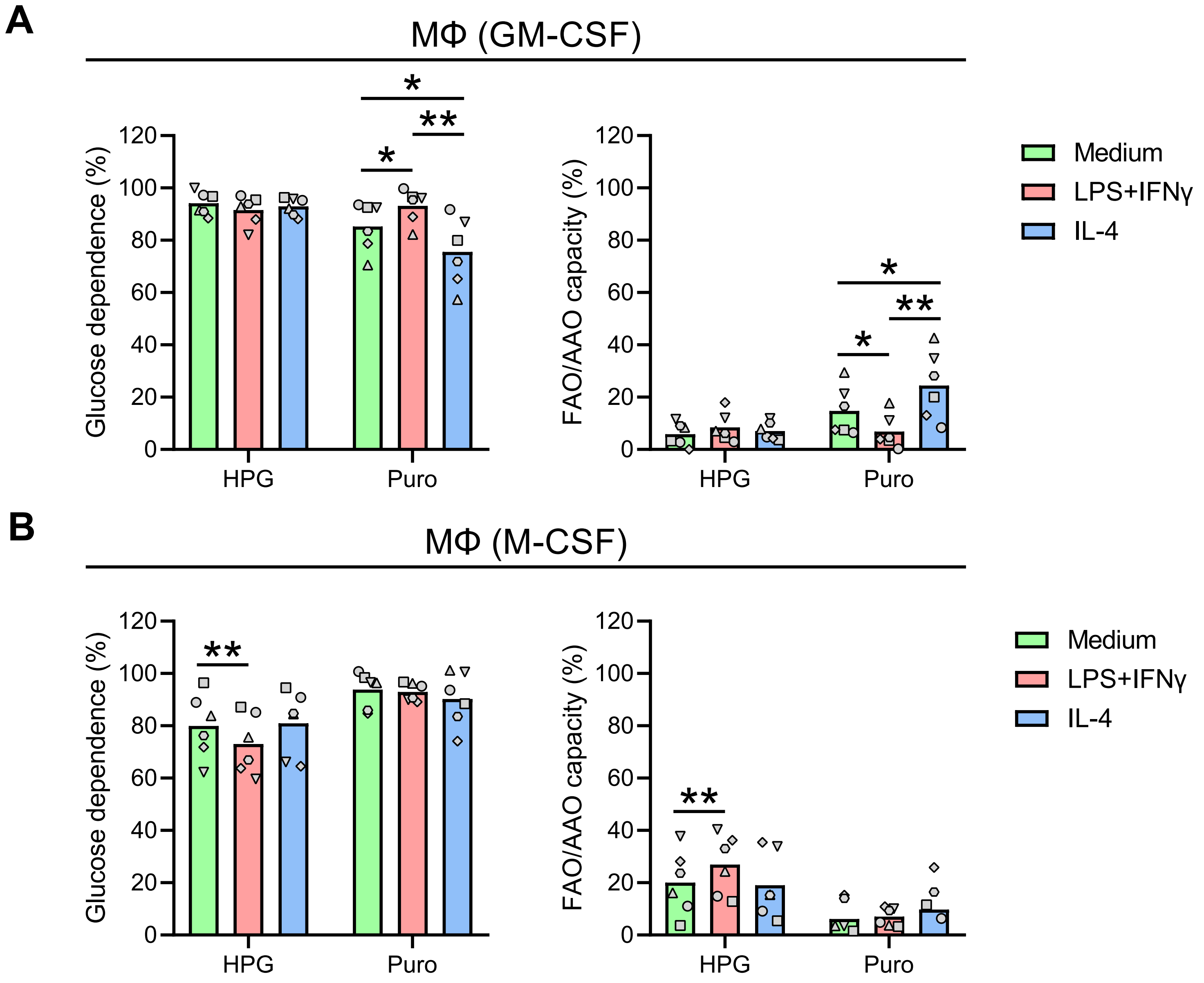
A
MΦ (GM-CSF)
Glucose dependence (%)
FAO/AAO capacity (%)
0
20
40
60
80
100
120
HPG
Puro
*
**
Medium
LPS+IFNγ
IL-4
B
MΦ (M-CSF)

Figure S1: Glucose dependence and FAO/AAO capacity of GM-CSF & M-CSF macrophages. Related to Figure 2. Primary human macrophages (GM-CSF and M-CSF) were stimulated for 24 hours with culture medium (green), LPS + IFNγ (red), or IL-4 (blue) before SCENITH analysis using either HPG or puromycin (Puro) as substrates. (A) Glucose dependence (%) and FAO/AAO capacity (%) of GM-CSF macrophages. (B) Glucose dependence (%) and FAO/AAO capacity (%) of M-CSF macrophages. Data are displayed as mean percentages ± SD (n=6). Significance was tested by Two-Way ANOVA with Sidak correction for multiple testing. Individual donors are displayed by different symbols. * = $p < 0.05$, ** = $p < 0.01$, *** = $p < 0.001$

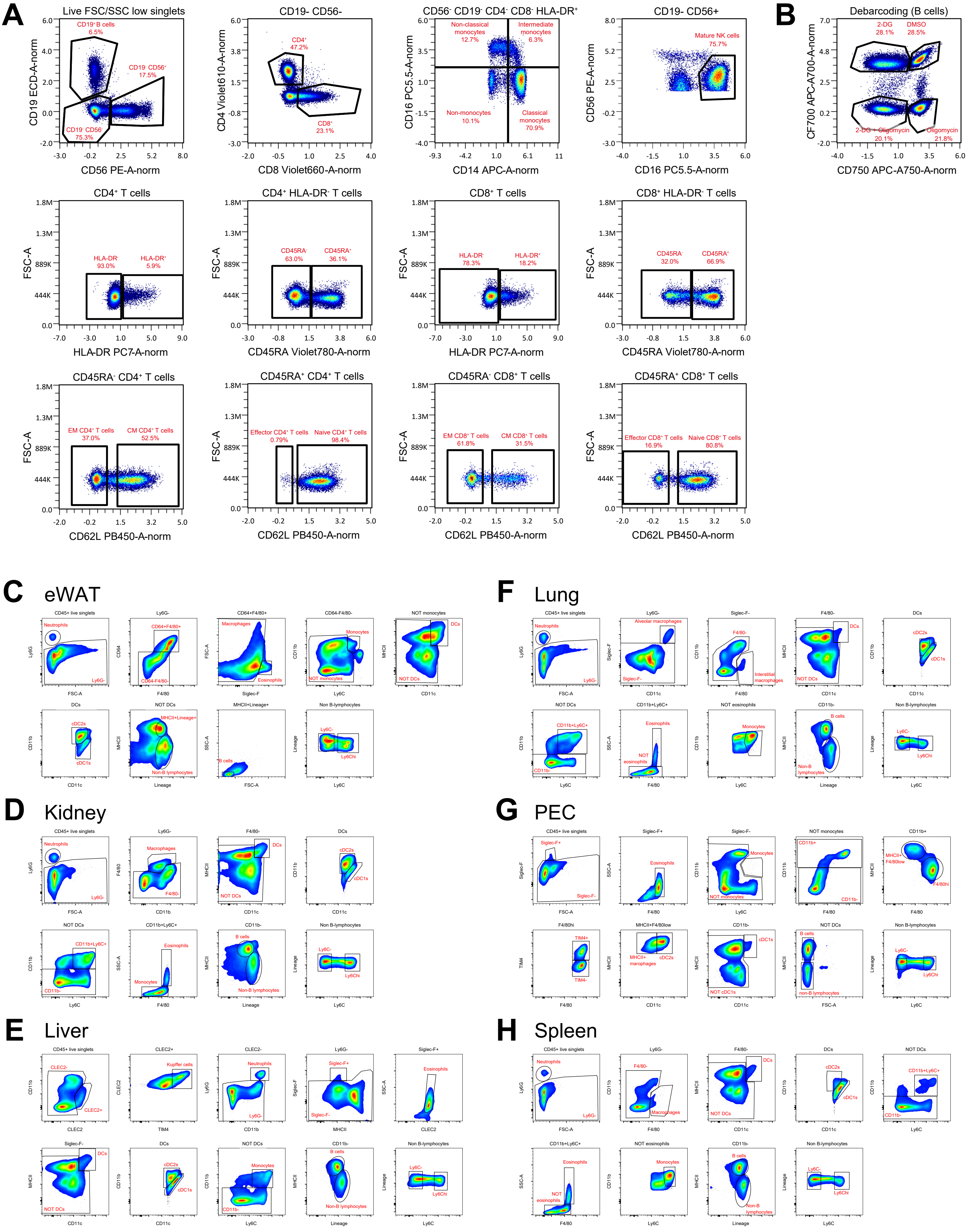
A
Live FSC/SSC low singlets
CD19⁺ B cells 6.5%
CD19⁻ CD56⁺ 17.5%
CD19⁻ CD56⁻ 75.3%
CD19 ECD-A-norm
CD56 PE-A-norm
CD19- CD56-
CD4⁺ 47.2%
CD8⁺ 23.1%
CD4 Violet610-A-norm
CD8 Violet660-A-norm
CD56⁻ CD19⁻ CD4⁻ CD8⁻ HLA-DR⁺
Non-classical monocytes 12.7%
Intermediate monocytes 6.3%
Non-monocytes 10.1%
Classical monocytes 70.9%
CD16 PC5.5-A-norm
CD14 APC-A-norm
CD19- CD56+
Mature NK cells 75.7%
CD56 PE-A-norm
CD16 PC5.5-A-norm
B
Debarcoding (B cells)
2-DG 28.1%
DMSO 28.5%
2-DG + Oligomycin 20.1%
Oligomycin 21.8%
CF700 APC-A700-A-norm
CD750 APC-A750-A-norm
CD4⁺ T cells
HLA-DR⁻ 93.0%
HLA-DR⁺ 5.9%
FSC-A
HLA-DR PC7-A-norm
CD4⁺ HLA-DR⁻ T cells
CD45RA⁻ 63.0%
CD45RA⁺ 36.1%
CD45RA Violet780-A-norm
CD8⁺ T cells
HLA-DR⁻ 78.3%
HLA-DR⁺ 18.2%
CD8⁺ HLA-DR⁻ T cells
CD45RA⁻ 32.0%
CD45RA⁺ 66.9%
CD45RA⁻ CD4⁺ T cells
EM CD4⁺ T cells 37.0%
CM CD4⁺ T cells 52.5%
CD62L PB450-A-norm
CD45RA⁺ CD4⁺ T cells
Effector CD4⁺ T cells 0.79%
Naive CD4⁺ T cells 98.4%
CD45RA⁻ CD8⁺ T cells
EM CD8⁺ T cells 61.8%
CM CD8⁺ T cells 31.5%
CD45RA⁺ CD8⁺ T cells
Effector CD8⁺ T cells 16.9%
Naive CD8⁺ T cells 80.8%
C eWAT
D Kidney
E Liver
F Lung
G PEC
H Spleen
CD45+ live singlets
Neutrophils
Ly6G-
CD64+F4/80+
CD64-F4/80-
Macrophages
Eosinophils
Monocytes
NOT monocytes
DCs
NOT DCs
cDC2s
cDC1s
MHCII+Lineage+
B cells
Non-B lymphocytes
Ly6C-
Ly6Chi
CD11b+Ly6C+
CD11b-
Alveolar macrophages
Siglec-F-
Siglec-F+
Interstitial macrophages
NOT eosinophils
Kupffer cells
CLEC2+
CLEC2-
TIM4+
TIM4-
MHCII+F4/80low
F4/80hi
NOT cDC1s

Figure S2: Gating strategies. Human PBMC 9-marker panel related to Figure 3: (A) Representative gating of monocyte subtypes, B cells, NK cells, CD4 T cells and subtypes, and CD8 T cells and subtypes. (B) Representative debarcoding gating (B cells). Gating strategy mice tissues related to Figure 4: representative gating of tissue-resident immune cell populations from (C) eWAT, (D) kidney, (E) liver, (F) lung, (G) PEC, and (H) spleen.

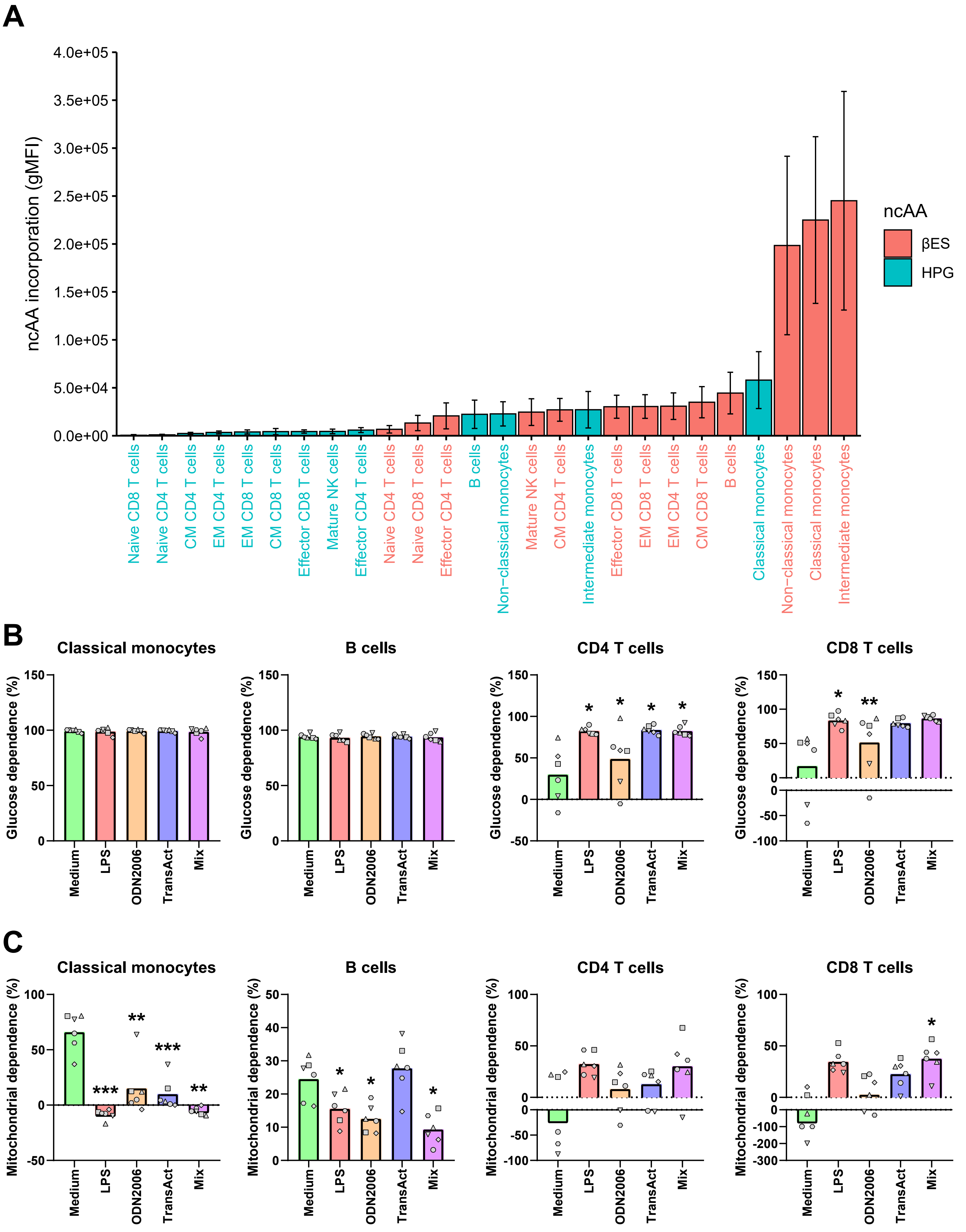
A
ncAA incorporation (gMFI)
4.0e+05
3.5e+05
3.0e+05
2.5e+05
2.0e+05
1.5e+05
1.0e+05
5.0e+04
0.0e+00
ncAA
βES
HPG
Naive CD8 T cells
Naive CD4 T cells
CM CD4 T cells
EM CD4 T cells
EM CD8 T cells
CM CD8 T cells
Effector CD8 T cells
Mature NK cells
Effector CD4 T cells
Naive CD4 T cells
Naive CD8 T cells
Effector CD4 T cells
B cells
Non−classical monocytes
Mature NK cells
CM CD4 T cells
Intermediate monocytes
Effector CD8 T cells
EM CD8 T cells
EM CD4 T cells
CM CD8 T cells
B cells
Classical monocytes
Non−classical monocytes
Classical monocytes
Intermediate monocytes
B
Classical monocytes
B cells
CD4 T cells
CD8 T cells
Glucose dependence (%)
Medium
LPS
ODN2006
TransAct
Mix
C
Classical monocytes
B cells
CD4 T cells
CD8 T cells
Mitochondrial dependence (%)
Medium
LPS
ODN2006
TransAct
Mix

Figure S3: CENCAT analysis of PBMCs using HPG. Related to Figure 3. PBMCs isolated from healthy blood donors (n=6) were stimulated for 2 hours with Medium control (green), LPS (red), ODN2006 (orange), TransAct (blue), or all stimuli combined (Mix, purple). (A) Relative incorporation (gMFI) of βES (red) and HPG (blue) in PBMC cell types under basal conditions (Medium control). (B) Glucose dependence (%) and (C) mitochondrial dependence (%) of classical monocytes, B cells, CD4 T cells and CD 8 T cells as determined by CENCAT using HPG as ncAA substrate. Significance was tested by Repeated One-Way ANOVA with Dunnett's multiple comparisons test. Individual donors are displayed by different symbols. * = $p < 0.05$, ** = $p < 0.01$

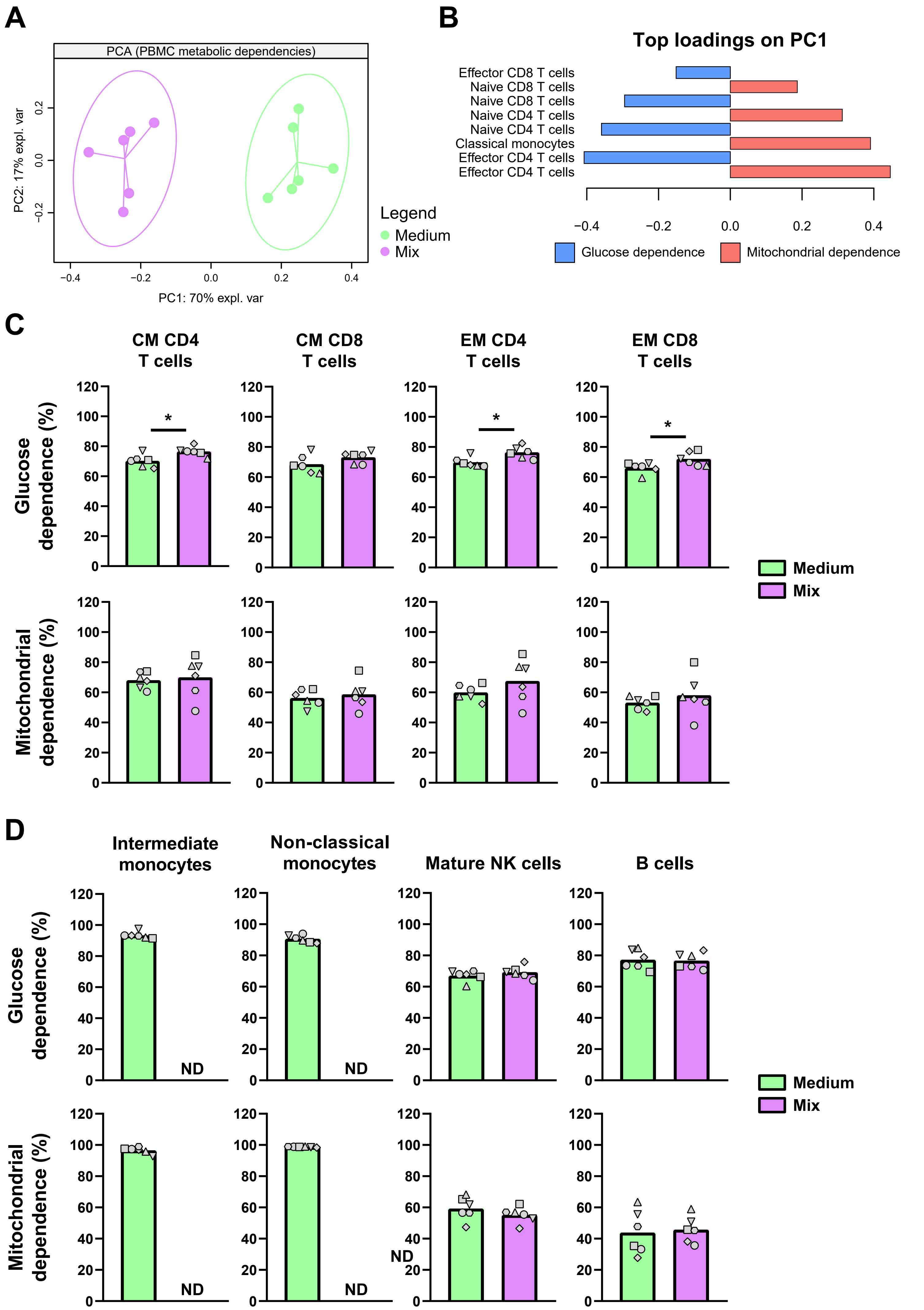
A
PCA (PBMC metabolic dependencies)
PC2: 17% expl. var
PC1: 70% expl. var
Legend
Medium
Mix
B
Top loadings on PC1
Effector CD8 T cells
Naive CD8 T cells
Naive CD8 T cells
Naive CD4 T cells
Naive CD4 T cells
Classical monocytes
Effector CD4 T cells
Effector CD4 T cells
Glucose dependence
Mitochondrial dependence
C
CM CD4 T cells
CM CD8 T cells
EM CD4 T cells
EM CD8 T cells
Glucose dependence (%)
Mitochondrial dependence (%)
Medium
Mix
D
Intermediate monocytes
Non-classical monocytes
Mature NK cells
B cells
Glucose dependence (%)
Mitochondrial dependence (%)
ND
Medium
Mix

Figure S4: CENCAT analysis of PBMCs using βES. Related to Figure 3. PBMCs isolated from healthy blood donors (n=6) were stimulated for 2 hours with Medium control (green) or complete stimulation Mix (LPS+ODN2006+TransAct, purple). CENCAT was performed using βES as ncAA substrate. (B) PCA score plot based on metabolic dependencies of PBMCs. (B) Top loadings on PC1 of the PCA score plot. Measures of glucose dependence are represented by blue bars and mitochondrial dependence by red bars. Glucose dependence and mitochondrial dependence (%) of (C) CM CD4 T cells, CM CD8 T cells, EM CD4 T cells and EM CD8 T cells, (D) intermediate monocytes, non-classical monocytes, Mature NK cells and B cells. Significance was tested by paired t-test. Individual donors are displayed by different symbols. ND = not detected. * = $p < 0.05$.

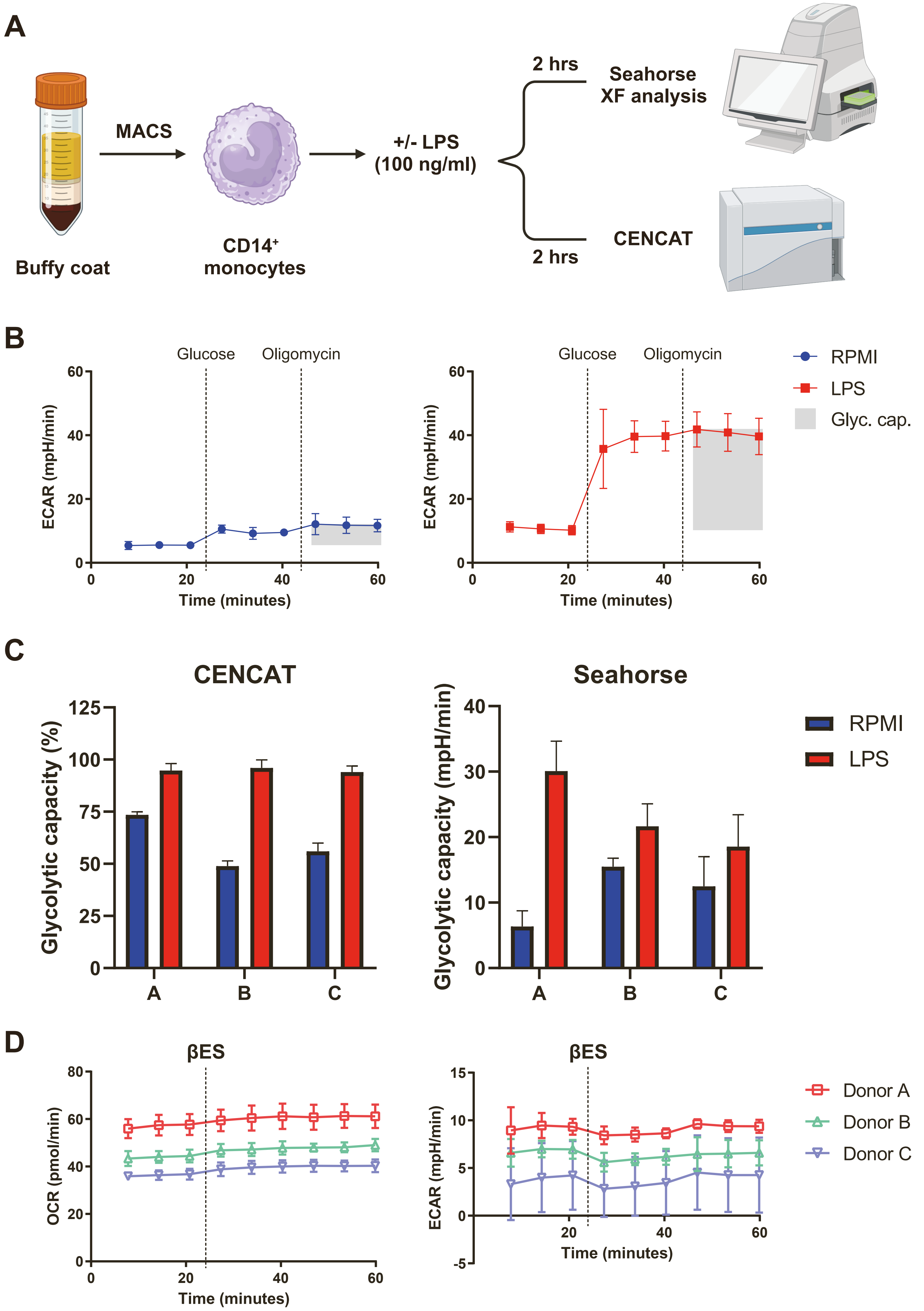
A
Buffy coat
MACS
CD14+ monocytes
+/- LPS (100 ng/ml)
2 hrs
Seahorse XF analysis
2 hrs
CENCAT
B
Glucose
Oligomycin
ECAR (mpH/min)
Time (minutes)
RPMI
LPS
Glyc. cap.
C
CENCAT
Seahorse
Glycolytic capacity (%)
Glycolytic capacity (mpH/min)
RPMI
LPS
A
B
C
D
βES
OCR (pmol/min)
ECAR (mpH/min)
Time (minutes)
Donor A
Donor B
Donor C

Figure S5: Comparison of CENCAT with Seahorse XF analysis. Related to Figure 3. (A) $CD14^+$ monocytes were isolated from buffy coats by MACS and either stimulated with LPS (100 ng/ml) or left unstimulated (RPMI) for 2 hours, after which cells were either measured using CENCAT or Seahorse XF. (B) Representative ECAR plots of RPMI (blue) and LPS (red) conditions after injections of glucose and oligomycin. Glycolytic capacity is depicted as a grey area under the curve. (C) Glycolytic capacity (%) as determined by either CENCAT or Seahorse XF for three buffy coat donors (A/B/C). (D)

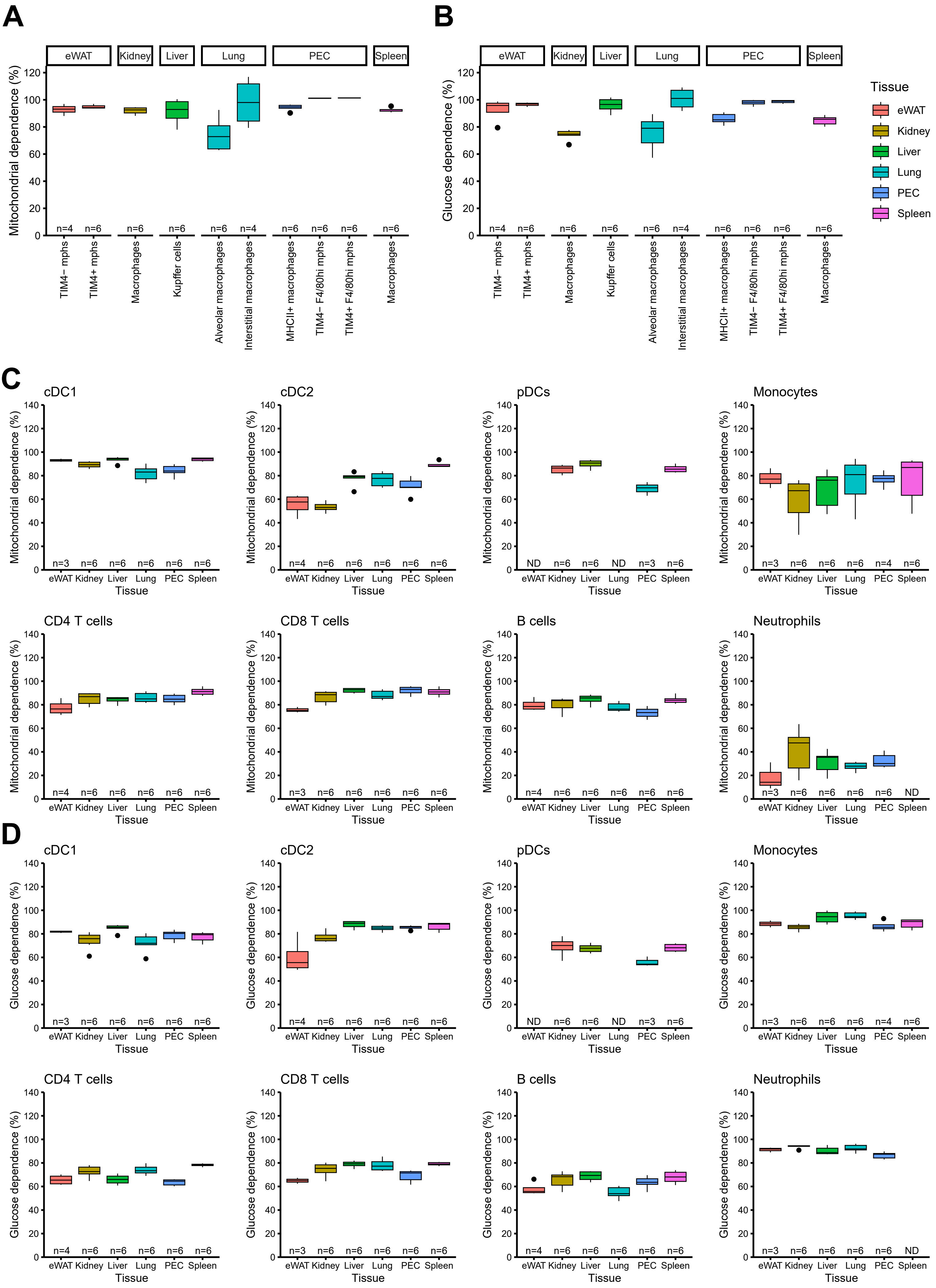
A
B
C
D
eWAT
Kidney
Liver
Lung
PEC
Spleen
Mitochondrial dependence (%)
Glucose dependence (%)
TIM4− mphs
TIM4+ mphs
Macrophages
Kupffer cells
Alveolar macrophages
Interstitial macrophages
MHCII+ macrophages
TIM4− F4/80hi mphs
TIM4+ F4/80hi mphs
n=4
n=6
n=3
ND
Tissue
cDC1
cDC2
pDCs
Monocytes
CD4 T cells
CD8 T cells
B cells
Neutrophils

Figure S6: Metabolic characteristics of murine tissue-resident immune cell populations. Related to Figure 4. The following tissues were isolated from male C57BL/6J mice and subjected to CENCAT analysis: eWAT (red), kidney (yellow), liver (green), lung (cyan), PEC (blue) and spleen (pink). (A) Boxplots of glucose dependence (%) and (B) mitochondrial dependence (%) of tissue-resident macrophage populations. (C) Mitochondrial dependence (%) of cDC1s, cDC2s, pDCs, monocytes, CD4 T cells, CD8 T cells, B cells, and neutrophils from all six tissues. (D) Mitochondrial dependence (%) of cDC1s, cDC2s, pDCs, monocytes, CD4 T cells, CD8 T cells, B cells, and neutrophils from all six tissues. Amount of samples (n) is indicated for each boxplot. ND = not detected.
